# How Genetics Can Improve Clinical Practice in Chronic Kidney Disease: From Bench to Bedside

**DOI:** 10.3390/jpm12020193

**Published:** 2022-01-31

**Authors:** Doloretta Piras, Nicola Lepori, Gianfranca Cabiddu, Antonello Pani

**Affiliations:** 1 Struttura Complessa di Nefrologia, Dialisi e Trapianto, ARNAS Brotzu, 09134 Cagliari, Italy; nicola.lepori@aob.it (N.L.); gianfrancacabiddu@aob.it (G.C.); antonellopani@aob.it (A.P.); 2 Dipartimento di Scienze Mediche e Sanità Pubblica, Università degli Studi di Cagliari, 09134 Cagliari, Italy; 3 Istituto di Ricerca Genetica e Biomedica (IRGB), Consiglio Nazionale delle Ricerce (CNR), 09042 Monserrato, Italy

**Keywords:** genetics, CKD, GWAS, precision medicine

## Abstract

Chronic kidney disease (CKD) is considered a major global health problem with high socio-economic costs: the risk of CKD in individuals with an affected first degree relative has been found to be three times higher than in the general population. Genetic factors are known to be involved in CKD pathogenesis, both due to the possible presence of monogenic pathologies as causes of CKD, and to the role of numerous gene variants in determining susceptibility to the development of CKD. The genetic study of CKD patients can represent a useful tool in the hands of the clinician; not only in the diagnostic and prognostic field, but potentially also in guiding therapeutic choices and in designing clinical trials. In this review we discuss the various aspects of the role of genetic analysis on clinical management of patients with CKD with a focus on clinical applications. Several topics are discussed in an effort to provide useful information for daily clinical practice: definition of susceptibility to the development of CKD, identification of unrecognized monogenic diseases, reclassification of the etiological diagnosis, role of pharmacogenetics.

## 1. Introduction

Chronic kidney disease (CKD) is considered a major global health problem with high socio-economic costs [1]. However, CKD is the definition of a complex phenotype, and encompasses a wide range of heterogeneous diseases. According to some authors, it may share some phenotypic traits with natural aging. Heritability estimates, linkage, and familial aggregation studies corroborate the contribution of genetics to both renal function in the healthy range and to kidney diseases [2,3]. The estimated heritability of GFR ranges from 44 to 75% [4,5,6], and from 4 to 18% for albuminuria [6,7,8]. A recent study conducted in the Netherlands estimated the heritability of renal function and other kidney traits in a 3-generation population of almost 156,000 individuals of European ancestry. The risk of CKD in individuals with an affected first degree relative was three times higher than in the general population, and their mean estimated glomerular filtration rate (eGFR) was lower compared to people of the same age [6].

## 2. Chronic Kidney Disease as a Complex Disease

The Genome wide association study (GWAS) is an approach used in genetics, and it is the preferred method for studying complex traits or diseases such as CKD. It involves scanning the genomes of a great deal of people who have a particular disease or condition, and of a likewise huge number of people who do not, and who are otherwise well-matched. The results are compared by statistical methods to identify differences that may contribute to the variability of a certain trait. Humans share about 99.9% of their genomes [9], and most of the variations appear to have no identified function, but a minority are associated with diseases. The differences observed in GWASs are usually genetic variants, in other words the substitution of a single base which is different from the reference sequence. Genetic variants represent the common variations in humans [10]; they are usually present in more than 1% of the population and are not necessarily related to a particular disease. Variants are classified as rare or common based on allele frequency; the extent of the difference between groups with a different variant is described as effect size. Variants with rare allele frequency and high effect size are usually associated with Mendelian diseases; however, complex traits or diseases are generally explained by a large number of common variants with low effect size [11]. GWAS is considered a hypothesis-free approach, and it is suited to identify associations between common genetic variants (minor allele frequencies >5% in the general population). With just one analysis more than 1 million genetic variants can be studied. The downside is that large samples are needed to identify variants which are rare (i.e., minor allele frequency, MAF <0.1) and/or with a low effect size (i.e., odds ratio 1.1–1.4) [12]. Moreover, since a considerable number of hypotheses are tested simultaneously, the probability of observing significant results due to chance is very high; methods to cope with multiple testing problems include the use of a very low *p*-value threshold (i.e., <5 × 10^−8^) and the need for large samples. To increase sample size and power, datasets are often combined with GWAS meta-analyses; by doing so, more variants are examined than in a single dataset alone.

Starting in 2010, several GWASs have been performed to identify which genetic variants are associated with eGFR variability [13,14,15,16,17,18]. To date, 309 genetic variants have been described as being associated with renal function; however, they account for only about 7% of its variability [19], even if estimated heritability for renal function is about 40%. The fraction of heritability not explained by the results of GWASs is defined as “missing heritability”. The explanations for this missing heritability include a much larger number of variants of smaller effect that are yet to be found, rarer variants (possibly with larger effects) poorly detected by available genotyping arrays, structural variants poorly captured by existing arrays, and inadequate accounting for shared environment among relatives [11]. Moreover, additional contributors to missing heritability may include gene–gene and gene–environment interactions, and mitochondrial or epigenetic inheritance.

Traditionally, two hypotheses have been proposed for complex diseases. According to the common disease common variant (CDCV) hypothesis, common allelic variants with low penetrance account for most of the genetic variance in disease susceptibility [20]. According to the other hypothesis (common-disease-rare-variant, [CDRV]), a large number of rare allelic variants with high penetrance account for the genetic variance in disease susceptibility [21].

In line with a modern view, CDCV and CDRV should not be seen as alternative hypotheses, but rather, they can explain the degree to which common and rare variations contribute to a particular disease phenotype [22]. Recent GWASs demonstrated that 309 loci are associated with eGFR variability in the general population. GWASs can usually detect relatively common variants (minor allele frequency >5%), but each of these loci have a very small effect, altogether explaining a low fraction of the eGFR variability [19]. The picture is now even more complicated thanks to more recent advances. As a matter of fact, although the more common alleles have been shown to have a small individual effect size, certain common alleles have a greater impact on disease risk, including HLA and PLA2R1 variants in membranous nephropathy (MN) [23].

Despite the achievements of GWASs, interpretation of results can be challenging. This is especially true in the case of neighboring loci in the disease-associated region which tend to be inherited as a unit, a phenomenon referred to as gametic association, or more commonly as linkage disequilibrium. The amount of linkage disequilibrium in different regions varies, and it is also influenced by factors other than recombination (i.e., population migration and admixture, population bottlenecks, and demographic history) [24]. A variant with the strongest association to the trait in GWAS cannot be the causal variant, therefore, it is necessary to perform studies which can help distinguish a causal variant from other variants that are only in linkage disequilibrium with it. A list of variants that achieve genome-wide statistical significance is used to determine regions of interest for fine mapping [24], which aims to define causal variants. The next step is to carry out functional studies, such as SNP enrichment methods to determine in which cell type the variants act, and colocalization to establish which genes are regulated by the variants [25]. 

## 3. Polygenic Risk Scores

Since each GWAS locus accounts for a very small fraction of overall risk, the translation of GWAS findings into clinical practice can be challenging. By aggregating the individual effect of a large number of variants, genome-wide polygenic risk scores (GPSs) attempt to offer a clinical tool. A GPS is a weighted sum of the risk alleles for a given condition, and the weight corresponds to estimates of its effect on disease risk from prior GWASs. It is interesting to note that in some circumstances the risk in individuals in the 1st and 2nd percentiles is the same as in individuals with rare monogenic diseases [26]. Vujkovic et al. proposed a GPS for the complications of type 2 diabetes mellitus [27]. Their multiethnic study includes almost 70,000 CKD patients; they found a strong association of GPS with some microvascular complications, such as retinopathy, while the correlation with CKD was modest. Interestingly, one of the genetic variants described was in the UMOD locus, whose mutations have been found both in monogenic diseases and associated with eGFR variability in CKD GWASs. 

This approach has been proposed in the field of nephrology to predict the risk of CKD. However, the genetic architecture underlying CKD is complex since it represents a highly heterogeneous group of diseases whose variability cannot be expressed by the common markers used in CKD-GWASs (i.e., serum creatinine, and eGFR). For these reasons, some authors propose that GPSs in nephrology should focus on single disease entities (i.e., IgA nephropathy) rather than on CKD [28].

A genetic risk score for MN has recently been proposed by Kiryluk’s group. In a relatively large GWAS, the multicenter study identified two new loci associated with MN. The proposed GPS explains 29% of the disease risk across the tested cohorts. Moreover, their research showed that combining the genetic risk score with anti-PLA2R ELISA allowed re-classification of 20–37% of cases in which the ELISA test was either negative or inconclusive. This approach could be proposed to make a diagnosis in a subset of patients in whom anti-PLA2R tested negative and kidney biopsy was not feasible [29]. 

## 4. *APOL1*: A Genetic Risk Factor

*APOL1* is an example of how knowledge of ancestry-specific alleles can have clinical utility for Mendelian nephropathies and for more widespread, complex forms of CKD. 

In people of African heritage, G1 and G2 coding variants of the *APOL1* gene are associated with non-diabetic end stage renal disease (ESRD) and contribute to nearly 70% of cases [30,31]. The *MYH9* gene, which encodes myosin-9, was initially thought to be a major genetic risk locus for a spectrum of non-diabetic ESRD. Tzur et al. demonstrated that *APOL1* (which is in almost perfect linkage disequilibrium with *MYH9*) was linked to the higher ESRD risk in African populations [30]. *APOL1* encodes apolipotrotein L1, a protein which is responsible for human innate immunity against *Trypanosoma brucei brucei*, thanks to its trypanolytic factors. However, two subspecies of Trypanosoma brucei (T. b. rhodesiense and T. b. gambiense) have managed to avoid this defense and thanks to their virulence factor called SRA, which inactivates the *APOL1* by binding it, they infect humans, causing sleeping sickness. Proteins encoded by one of the two *APOL1* variants (G1 or G2) can escape this binding and therefore maintain their activity against T. b. rhodesiense and T. b. gambiense, thus providing a (dominant) selective advantage [32]. *APOL1* risk variants arose approximately 4000 years ago in Africa. The ancestors of modern Europeans left Africa several millennia earlier, so the risk alleles are not found in Europeans [32]. On the other hand, risk alleles can be detected in more than 30% of African Americans, and G1 was found in about 40% of Yoruba, in Nigeria [31]. In the AASK cohort, about 60% of patients who were homozygous for the risk variants (either G1 or G2) reached the composite renal outcome (doubling of serum creatinine level from baseline or incident ESRD) during follow-up, and the risk of reaching the outcome was about double compared both to patients who were heterozygous for the variants and those who carried the reference sequence [33]. The analysis of the CRIC cohort extended these results to patients affected with diabetes, using a Caucasian cohort as the comparison group: among the patients with diabetes the decline in eGFR was−1.5 mL/min/1.73m^2^ per year in Caucasian patients, versu -2.7 in African ancestry patients in the *APOL1* low-risk group, and−4.3 in African ancestry patients in the ApoL1 high risk group, while it was analogous in non-diabetic patients, (respectively: −0.7, −1.0, −2.9). In two CKD cohorts, *APOL1* risk genotypes were found in 29% of African ancestry patients and in 7% of Hispanic patients, and they were frequently found in patients with a diagnosis of nephropathy of unknown origin (24%) [34]. 

Susceptibility to different kinds of renal diseases is largely increased by *APOL1* risk variants, with the highest risk for HIV nephropathy (odds ratio, [OR] 29–89), FSGS (OR 17), and hypertensive nephropathy (OR 7–11). For this reason, it has been proposed that these diseases may be included as part of an *APOL1* nephropathy spectrum rather than being considered distinct entities in individuals with the high-risk genotype [35].

Despite the increased risk of kidney disease, the frequency of *APOL1* risk variants in people of African ancestry is high. This can be explained by a heterozygous advantage model, since the association with renal disease is recessive, while the protective effect against T. b. rhodesiense is dominant [31].

*APOL1* status may have implications for living and deceased donor selection. Recipients of *APOL1* high-risk kidneys may have shorter allograft survival duration, with a higher failure rate than what is observed in recipients from donors carrying zero or one *APOL1* risk allele [36].

Doshi et al. compared a group of 19 living donors carrying two *APOL1* renal risk alleles to 117 carrying zero or one *APOL1* renal risk allele. In the *APOL1* high risk group the pre-donation and post-donation eGFRs were lower than in the low-risk group [37]. However, differences of opinion exist among nephrologists concerning whether living donors of African ancestry should be tested for *APOL1.* To address this question, a new trial has been designed. The *APOL1* Long-term Kidney Transplantation Outcomes Network (APOLLO) aims to enroll 2614 deceased-donor recipient pairs, as well as some living kidney donor-recipient pairs and some unpaired deceased-donor kidneys; all the donors must be of African ancestry [38].

## 5. How Genetics Can Support the Clinical Setting in CKD

Hereditary kidney diseases include a series of rare diseases; however, they cause 10% of ESRD in adults, and the percentage of ESRD patients reporting a family history of CKD is even higher. This number is extraordinarily elevated in the pediatric setting, where at least 70% of cases of ESRD are estimated to be secondary to genetic diseases [39].

Genetics can be employed in several ways in clinical settings. When used with more traditional tools, it can confirm, reclassify or establish a new diagnosis. It can be used in reproductive planning as well. It may help identify a certain disease within a family and avoid potentially unnecessary testing in other family members. The knowledge of a genetic disease in a potential transplant recipient can improve patient management and provide more information on the prognosis (i.e., recurrence in patients with Fabry disease or hyperoxaluria; autoantibodies in patients with COL4A5 mutations); genetic testing can also be used to assess young, related kidney donor candidates.

## 6. Diagnosis/Reclassification

Although not infrequent, hereditary forms of renal diseases may be difficult to distinguish from acquired forms unless more specific diagnostic tools are used. In the era of precision medicine, genetic tests can be very useful tools to identify the cause of nephropathy of unknown origin, since management and prognosis can be very different. The absence of family history does not exclude the possibility of a genetic disease; this is especially true in the case of autosomal recessive diseases. Genetic disorders involving the kidneys are heterogeneous. Several chromosomal abnormalities are associated with renal diseases. Trisomy 21 can be associated with hydronephrosis, multicystic kidneys, obstructive lesions, and renal agenesis; monosomy X may be associated with horseshoe kidney and duplication, as well as vascular abnormalities. Congenital anomalies of the kidney and urinary tract (CAKUT) syndrome encompasses a broad spectrum of phenotypes and can be characterized by multicystic, dysplastic, or hypoplastic kidneys, renal agenesis, ectopic kidney, reflux, or hydronephrosis. To date, mutations in over 50 genes have been described in isolated and non-isolated CAKUT [40,41].

Traditionally, Mendelian diseases are described as rare, caused by variants with large effect sizes, and associated with significant morbidity. Different variants in some genes that are known to cause Mendelian disease can also contribute to complex disease. Genetic variants in Mendelian loci (i.e., UMOD) have been reported as risk factors for CKD in GWASs.

According to some authors, CKD might represent a wide array of rarer disorders, each of which has its own distinct genetic architecture [42].

In a seminal work led by Ali Gharavi, exome sequencing was carried out in 3315 patients with CKD, mostly adults, many of whom had ESRD. Surprisingly, 9.3% of the patients turned out to have a genetic disease. The highest percentage of monogenic diseases was found in patients identified as having “congenital or cystic disease” (diagnostic yield 23.9%). Interestingly, among the patients with nephropathy of unknown origin, a genetic defect was found in 17.1% of them, with a broad range of mutations, encompassing defects of collagen (either *COL4A3*, *COL4A4*, *COL4A5*), UMOD-associated tubulointerstitial disease, mutations in PKD1, and other less frequent defects. Nephropathy of unknown origin and congenital or cystic diseases were shown to be independent predictors of having a genetic diagnosis. The diagnostic yield was lower in the other renal phenotypes: 7.2% in patients with glomerulopathy, 4.5% in those with tubulointerstitial disease, and 2.5% and 1.6% in patients with hypertensive and diabetic nephropathy, respectively. Of the 66 monogenic diseases described, 6 accounted for 63% of the genetic diagnoses. Mutations in PKD1 or 2 were described almost exclusively in patients with congenital or cystic disease; COL4A–either 3, 4 or 5- were prevalently found in renal diseases defined as “glomerular”, while UMOD mutations were found in patients with a more heterogeneous renal phenotype [34].

The diagnostic yield in certain glomerular diseases can be even higher. Yao et al. performed whole exome sequencing in a cohort of 193 individuals with either a histological diagnosis of FSGS or proteinuria and a relative with FSGS, only 14 of whom where related to some other participant. Interestingly, in 11% of cases a genetic cause was found; additionally, another 9% of patients carried a likely pathogenetic mutation. More than half of the pathogenic cases were due to a mutation in *COL4A3/4/5*; 40% in genes encoding important podocyte proteins and 5% in CAKUT. It is remarkable that the mean age was 34 years (±16), changing the old paradigm of renal genetic diseases affecting only children [43].

In the last decade, a high number of new mutations have been described as causative of steroid-resistant nephrotic syndrome.

Wang et al. conducted sequencing of 662 whole exomes from 363 unrelated families (including a total of 483 affected individuals) and as many healthy controls. The analysis validated many of the already known FSGS-causing genes in FSGS patients. Most interestingly, it showed that the prevalence of some additional candidate FSGS was higher in controls, highlighting how the genetic diagnosis of FSGS can be complicated by the non-negligible percentage of variants in FSGS-related genes in healthy individuals. Some variants of genes that are usually associated with other renal phenotypes can present a FSGS pattern, probably as a response to primary injury. This can be the case with UMOD variants or CLCN5, usually associated with tubular dysfunction, or the mutations in type IV collagen genes and nephronophthisis genes [44]. An explanation may be that, while most cases of adult-onset familial FSGS are inherited as an autosomal dominant disease, penetrance is often incomplete with variable expression [45].

In a cohort of 1783 unrelated families with a diagnosis of FSGS, Sadowski et al. performed whole exome sequencing of NPHS2 and WT1, and Sanger sequencing for the FSGS genes described in the literature. The diagnostic yield decreased at a very high rate depending on the patients’ age at onset, starting from 60% in newborns, to about 5–10% in teenagers. Remarkably, some variants were typically associated with early onset nephrotic syndrome (NS), while others with late onset NS [46].

The analysis of 70 families with FSGS conducted by Malone et al. showed that 10% had variants in *COL4A3* and *COL4A4*, which are involved in Alport syndrome and thin basement membrane nephropathy [47]. All these studies prove that the addition of genetic analysis will help further characterize many histological findings.

A paper written by Jayasinghe et al. aimed to explore whether and how a genetic diagnosis could change the clinical management of patients. They enrolled a cohort of 204 patients with a suspected monogenic disease, prioritizing those with at least one of these characteristics: family history of renal disease, syndromic features, or childhood onset of disease. They were categorized according to their phenotype, and a first line of tests was carried out on the basis of this category (i.e., cystic panel for patients with renal cystic disease). If there were no results, analysis was expanded to a broader group of 336 known kidney disease genes. When a syndromic diagnosis was suspected, a virtual panel of about 4000 genes was tested. Eighty patients received a diagnosis of monogenic disease; 51 had a clinically relevant variant of uncertain significance and 73 had a negative result. Among the patients with a positive result, diagnosis was confirmed in 34%, it was clarified in 28%, and it was reclassified in 39% of cases. The genetic diagnosis ruled out the need for a renal biopsy in 13% of the cases, while it changed the clinical surveillance in 44% of patients, and the treatment plan in 20% [48].

## 7. When to Ask for a Genetic Test for Monogenic Kidney Diseases

Several clinical factors can predict the diagnostic yield: the presence of extra renal features, young age at onset, family history of CKD, and the kidney disease subtype [49]. Consequently, a genetic test may be useful when a phenotype can be explained by a monogenic disease and/or when the patient has multiple symptoms. It is intuitive to look for a genetic disease when several relatives are affected; however, in pediatric patients and young adults (≤40 years) a test can be useful regardless of family history since sporadic cases may be due to spontaneous pathogenic variants (de novo) or autosomal recessive disease. Finally, some patients may benefit more from a genetic test based on their clinical phenotypes (i.e., cystic disease), as might individuals for whom a cause of CKD has not been found despite a thorough workup.

It should be emphasized that sometimes the phenotype can be atypical even in Mendelian diseases, and this may be explained by several factors. The same disease can be caused by different genes, and this could influence the outcome (i.e., mutations in PKD1 are associated with earlier ESRD than PKD2 in autosomal dominant polycystic kidney disease [ADPKD]); this is known as genetic heterogeneity. On the other hand, allelic heterogeneity means that different mutations in the same gene give rise to a different phenotype. Other factors, such as incomplete penetrance, mosaicism, somatic mutations, X-inactivation, and more complex models of inheritance (i.e., mutations in more than one gene), can complicate the interpretation of phenotype [50,51].

A factor that needs to be considered before ordering a genetic test is whether or not the results will alter the clinical management of the patient. For example, in ADPKD, genetic tests are not routinely prescribed since -in most cases- the diagnosis can be clinical, and the outcome models rely on imaging. In this setting, a genetic test should be ordered in selected cases, i.e., when ADPKD is suspected in the absence of family history or when equivocal imaging findings are found, or to screen young, potential high-risk kidney donors, or to come to a prenatal and pre-implantation genetic diagnosis [52].

## 8. Sequencing Targets

Nowadays, several genetic tests are available, but regardless of the test that is chosen, it must be targeted to the patient, thus close interaction between the clinician and the laboratory is required [53].

Traditionally, diagnostics in monogenic diseases has been based either on single gene sequencing, and this methodology is still being used for specific disorders caused by the mutation of a single gene (i.e., ADPKD genes), or on a targeted panel, which allows to study many genes in the same analysis for more heterogeneous disorders with well-defined disease-associated genes (i.e., Alport disease). Recently, some “kidney gene panel” tests have become available, i.e., Renasight^®^ (natera^NT^, Austin, TX, USA), which tests 385 genes associated with kidney disease. One step further is whole exome sequencing, which studies all the expressed RNA and comprises 1% of the whole genome. The next step could be whole genome sequencing, which encompasses both coding and non-coding regions.

One important point is that the read depth changes depending on the methodology we use and on the amount of genetic material we are studying. If we look at a single gene, the amount of read is much deeper; however, in a targeted panel, the amount of depth decreases, while in exome and whole genome sequencing the depth decreases even further.

Another point to consider is that the more information we have, the more the background noise increases. Every individual carries thousands of rare variants, but only a minority of these variants cause rare genetic disorders. When a variant is described in a genetic test, it is important to establish whether it is a disease-causing gene. According to guidelines, variants are classified as “pathogenic,” “likely pathogenic”, “likely benign,” or “benign”. When they do not fit in any of these categories, they are described as “variants of uncertain significance”, which are very frequent; their definition is not static, since it can change when new knowledge about that variant is acquired. The interpretation of these results is based on the kind of variant (some of which are assumed to disrupt gene function, i.e., nonsense and frameshift mutations), minor allele frequency in the general population, and on co-segregation analysis [54]. It is important to know that finding a mutation in a gene that is definitely a disease-causing gene is not equivalent to diagnosing a genetic disease in a particular patient because there may be variants which are defined as “benign”. This can be particularly challenging for dominant diseases, while for recessive diseases it is usually easier since the patient carries the same variant in both alleles of a gene. Guidelines recommend the use of ancestry-matched controls to interpret variants, since some previously reported pathogenic variants have been reclassified as benign or disease-predisposing due to their high frequencies in some populations [55]. A recent study, carried out in two European cohorts, showed that each tested individual carried on average 2.0–2.3 pathogenic or likely pathogenic variants for autosomal recessive genes, and 1.1–1.5 for recessive genes [56].

Depending on the patient’s condition and his/her family history, the clinician should choose which test is best. For example, if another family member already has a genetic diagnosis, or when one gene or mutation accounts for the majority of the patient’s clinical presentation, potentially the best approach is based on single gene sequencing; however, when the cause of the disease is not known, whole exome or whole genome sequencing should be the preferred method. In some cases, it may be necessary to switch-from a single gene to a targeted panel, or to a more extensive analysis, if the narrow analysis has not provided any results.

## 9. Drawbacks

Although whole genome sequencing is increasingly being used to help diagnose rare diseases, some concerns have been raised regarding its extensive incorporation in clinical practice. According to some experts, laboratories may not have adequate resources to deal with a high number of requests; this may translate into long turn-around times for a report. Moreover, the training clinicians receive may not suffice to disclose the results to a patient, to interpret them or to fully understand the limitations of a test [57].

Improvements in technology have resulted in a significant decrease in the cost of genetic testing: in the last decade, the cost of genome sequencing dropped from more than US$7000 to about US$500, and it is expected to continue to decrease in the future [58]. Full multigene panels for hereditary cancers and/or cardiovascular disease may be available for $250 to $350. However, when used on a large-scale, the cost is still high. Moreover, the interpretation of DNA test results is expensive. Some authors have reviewed the cost-effectiveness of precision medicine [59] and found that it can be influenced by many factors, including the accuracy of a genetic test, its sensitivity and specificity. False-positive and false-negative tests might result in unnecessary treatments and in increased morbidity and mortality, and this should be kept in mind when a new test is prescribed.

## 10. Pharmacogenomics

Pharmacogenomics is the study of how genes affect a person’s response to pharmacologic agents. Genetic factors can influence the response to drugs thereby affecting the pharmacokinetics, the pharmacodynamics, and predisposing to idiosyncratic reactions or conditioning the response of a disease to a certain drug. Migalastat is a chaperone therapy used in Fabry disease; it selectively binds and stabilizes otherwise unstable α-galactosidase A enzymes, allowing their normal trafficking and thus increasing enzyme activity in lysosomes. In the USA, to be eligible for this treatment a patient must have a migalastat-amenable variant of GLA that is interpreted as pathogenic or likely pathogenic [60,61].

Azathioprine is an antimetabolite used for maintenance therapy in some types of nephritis and in post-transplantation. It is converted to the active mercaptopurine, which is then methylated to inactive metabolites through polymorphic thiopurine methyltransferase (TPMT). Individuals with reduced TPMT activity are exposed to higher levels of active metabolites, and are at high risk of side effects, including severe bone marrow suppression. TPMT activity is influenced by genotype, and near-zero TPMT activity occurs in about 0.3–0.5% of the general population. The use of genetic testing has been proposed to screen patients before prescribing azathioprine. Azathioprine should be avoided in patients who inherit two nonfunctional *TPMT* alleles, while in heterozygous individuals a lower initial dosage should be given [62].

Interestingly, the use of pharmacogenomics has been proposed for the selection of patients in clinical trials, i.e., in FSGS/minimal change studies to exclude individuals with genetic nephrotic syndrome from corticosteroid trials [63].

Pharmacogenomics may be a useful tool, but several barriers prevent its implementation in everyday clinical practice, including test availability and costs [64].

## 11. Genetics and Development of New Drugs for Renal Diseases: A Future Prospective

The gap between basic research and clinical practice is often referred to as the “valley of death” [65]. One of the challenges of genetic studies has been the functional interpretation of genes and genetic variants. The translation into therapeutic targets and drugs can be even more arduous. One of the main obstacles is inferring the causal genes from the GWAS results, which can then be used as drug targets. This is especially true for the non-coding variants because determining their effect can still be problematic. Despite the difficulties, GWASs seem to offer a striking opportunity in this field, as shown by Okada et al. In a GWAS meta-analysis involving more than 100,000 individuals of European and Asian ancestry, it was shown that 98 rheumatoid arthritis candidate genes were the targets of approved therapies for this disease [66]. Moreover, it has been proved that drugs with genetically supported targets were more likely to be successful in phase II and III trials, especially when the causal genes were clear [67,68]. It is remarkable that human genetic studies on the role of PCSK9 in modulating LDL cholesterol are based on the discovery of PCSK9 inhibitor monoclonal antibodies [69]. Since the discovery of a new drug and the process leading to its approval can be highly time- and money-consuming, an appealing approach is to repurpose drugs already approved for other indications, as reviewed elsewhere [70]. This may be promising in the field of renal diseases and CKD [71].

In summary, genetics provide clinicians with very useful tools which, together with more traditional diagnostic methods, can be used to diagnose, reclassify or better define some medical conditions. Moreover, it can support the selection of patients who may benefit from a specific treatment, avoiding adverse effects in those who would surely not respond. The aim is to have more personalized and precise medicine and thus to improve patient care.

## Data Availability

Not applicable.
